# Assessment of the Feelings and Attitudes of Children towards Their Dentist and Their Association with Oral Health

**DOI:** 10.1155/2014/867234

**Published:** 2014-03-27

**Authors:** Aslı Patır Münevveroğlu, Beyza Ballı Akgöl, Tuğba Erol

**Affiliations:** Department of Pedodontics, Faculty of Dentistry, Istanbul Medipol University, 34083 Istanbul, Turkey

## Abstract

This study assessed the feelings and attitudes of children toward their dentists and their association with oral health. *Methods.* A questionnaire designed to evaluate the attitudes of children toward dentists and preferences was completed by 200 children and their families. In addition, the children were examined and the World Health Organization (WHO) method was used to obtain dmft and DMFT scores. *Results.* The mean dmft and DMFT scores were 3.52 ± 2.44 and 1.35 ± 1.29, respectively. Of the children, 92.5% reported that they had visited a dentist before. Of the children who had visited dentists, approximately 71.5% reported that they enjoyed their visits. Of the children, 84% preferred female dentists, 76.5% preferred dentists that wore a colored coat, and 63% preferred a decorated dental clinic over a plain clinic. The mean dmft and DMFT values of children who were afraid of dentists were significantly higher than those of children who were not (*P<*0.01 and *P<*0.05, resp.). *Conclusion.* Children have strong perceptions and preferences regarding the appearance of their dentists and dental clinics. The results of this study might help dentists to improve their delivery of care.

## 1. Introduction

In dental practice, most children do not cooperate during dental procedures and sometimes it is very difficult to manage a child in a dental clinic. These management difficulties are related not only to the technical procedures involved, but also to the different emotional upsets experienced by children. The most common emotional upsets seen during dental treatment are anxiety and fear, which might originate from a previous traumatic experience in the dental office or during hospitalization for other reasons [1, 2].

Dental anxiety and fear of dental treatment in children are recognized in many countries as a public health dilemma [3]. The terms dental fear and dental anxiety are often used synonymously [4] and are considered to be the main reason for behavior management problems and avoidance of dental care [5]. These problems sometimes require replacement of conventional treatment with more complicated alternatives, such as sedation or general anesthesia [6]. Surveys indicate that 5-6% of the population and 16% of school-age children fear dentists [7]. Indeed, children with active caries are more afraid than other children due to negative treatment experiences [8]. Alvesalo et al. reported that boys with a DMFT score ≥1 had a significantly higher mean dental fear than those who were caries-free [9].

It is important for dentists to establish a friendly relationship with patients, especially children, to reduce patient fear and achieve efficient dental treatment. Children who have positive interactions with their dentist will be less likely to develop a fear of dentists and will experience less anxiety. As a result, they will be more likely to visit the dentist and will have better dental health [7].

We assessed feelings and attitudes of children toward their dentists and their association with oral health.

## 2. Materials and Methods

Data were obtained from a sample of children using a questionnaire developed by AlSarheed [7]. The study protocol received institutional approval from the Ethics Committee of Istanbul Medipol University, Istanbul (B.30.2.İMÜ.O.05.05-GOKAEK-05). The study group comprised children aged 6–12 years attending the Department of Pedodontics, Faculty of Dentistry, Istanbul Medipol University for routine followup. The questionnaire was administered to 200 children, comprising 98 (49%) girls and 102 (51%) boys. Consent was obtained from parents/guardians to participate in the study. 200 children were requested to participate in the study to obtain 200 completed questionnaires. The response rate was 100%.

The questionnaire included seven items designed to assess children's perceptions of their dentists. The subjects were asked if they had visited the dentist before and if there were any physicians in the family. They were also asked to describe the attitudes of siblings regarding dental visits. In addition, each child was asked to choose pictures that indicated their preference regarding dentist gender, attire, and protective equipment, and the design of the dental clinic.

The clinical examinations were performed by one examiner (TE) using disposable mouth mirrors for indirect vision of the lingual areas of the teeth and torchlight. A community periodontal index (WHO-BPE probe/G21) probe was used to confirm visual evidence of caries on the occlusal, buccal, and lingual surfaces. During the examination, the children were seated on a chair. Gauze pads were used to clean and dry the teeth surfaces before the examination. A schedule for data collection was prepared in advance and 40–50 children were examined each day. A dental surgeon sat close to the examiner to hear and record the codes. The World Health Organization (WHO) criteria (1997) were used to diagnose caries [10].

### 2.1. Statistical Analysis

The questionnaire data were coded and entered into SPSS ver. 15.0 (SPSS, Chicago, IL, USA) for statistical analysis. The conformation of parameters to normal distributions was evaluated using the Kolmogorov-Smirnov test. Descriptive and analytic approaches were used in the data analysis. The chi-square test, Mann-Whitney *U* test, and Student's *t*-test were used to determine relationships among variables. Spearman's rho correlation was used to evaluate parameters that were distributed normally.

## 3. Results

The study group comprised 200 children aged 6–12 (mean 9.41 ± 1.48) years attending the Department of Pedodontics, Faculty of Dentistry, Istanbul Medipol University for routine visits, including 98 (49%) girls and 102 (51%) boys. The mean dmft and DMFT scores were 3.52 ± 2.44 and 1.35 ± 1.29, respectively.

Of the study children, 92.5% had visited a dentist before. The children's responses to their previous dental experience are summarized in Table 1. The majority of children (71.5%) reported that they enjoyed their visit to the dentist. The perceptions of children who had a physician in the family were significantly different from those of children who did not. Approximately 26% of the children reported that a sibling had a pleasant perception of their visit to a dentist.

The children's perceptions of their dentists are summarized in Table 2. Approximately 84% of the children preferred to be treated by a female dentist; 76.5% preferred their dentist to wear a colored coat instead of a white one. This preference was significant. Of the children, 20% preferred their dentist not to wear protective equipment. When shown pictures of the same dentist wearing a mask or protective eye glasses or both the mask and eyewear, 8.5% of the children picked the picture of the individual wearing both pieces of protective gear as the dentist they would like to be treated by, while 1% chose the picture of the dentist wearing eye glasses and 70.5% chose the picture of the dentist wearing a mask.

When the children were asked to choose between pictures of undecorated and decorated dental clinics as the clinic they would like to be treated in, 76.5% selected the decorated dental clinic. However, there was no significant difference between the age groups (*P* < 0.05).

Children described several causes of fear related to visits to the dentist's office. These fears were related to injection (60%), tooth extraction (50%), restorations (3.6%), and the sight of dental instruments (40%).

The children who were afraid of the dentist were significantly younger than those who were not (*P* < 0.01). There was no significant difference in the mean age of the children according to dental clinic perception (*P* > 0.05) (Table 3).

The distributions of the dmft and DMFT scores by gender and fear of dentists are shown in Table 4. There was no significant difference between the mean dmft and DMFT scores according to gender (*P* > 0.05). The mean dmft and DMFT scores of children with dental fear were significantly higher than those of those who did not (*P* < 0.01, *P* < 0.05).

In this study, 84% of the children preferred to be treated by a female dentist; this was not affected by the gender of the child (*P* > 0.01) (Table 5).

## 4. Discussion

Sitting on a dental chair under bright lights and hearing the noise coming from the equipment can be an unpleasant experience for children. A pediatric dental office should be designed in such a way that children can feel comfortable as they wait for their appointment. The children in this study favored a decorated dental clinic over a plain clinic. This concurs with the report by AlSarheed, in which 63% of the children selected the decorated clinic [7].

Physical appearance is a factor in a person's choice and plays an important role in the development of the physician-patient relationship. Mistry and Tahmassebi assessed the attitudes of children and parents towards dental attire and found that parents favored traditional dress as it gives an air of professionalism [11]. AlSarheed indicated that children prefer their dentist to wear the traditional formal attire with a white coat as they see it as a symbol of healing [7]. This finding supports the report by McCarthy et al. [12] who found that children are not afraid of a physician in a white coat. In our study, however, 76.5% of the children preferred their dentist to wear a colored coat instead of white one.

The majority of the children in this study reported that they liked their first visit to dentist, which is consistent with the results of AlSarheed [7] and Mittal and Sharma [2]. Mittal and Sharma [2] indicated that younger children had more negative perceptions than older children because younger children could not comply satisfactorily. Klein [13] and Oppenheim and Frankl [14] reported similar findings. Similarly, the children who were afraid of a dentist were significantly younger than those who were not.

Dental anxiety is likely predictive of the occurrence of dental caries. We found a significant difference between dmft/DMFT values and dental fear; the mean dmft and DMFT scores were significantly higher in children who had dental fear (*P* < 0.01, *P* < 0.05). Conversely, Taani et al. [3] found no correlation between “general dental fear” and DMFT scores.

Mistry and Tahmassebi [11] reported a significant difference in the preference of the participants for the gender of their dental health care provider: male participants favored male students and females preferred female students. In our study, 84% of the children preferred to be treated by a female dentist, regardless of the child's gender.

Proper dress and the use of protective clothing protect both patients and health care providers from infectious diseases. We found that 70.5% of the children preferred that dentists wear masks. Similarly, Shulmam and Brehm [15] reported that 70% preferred that dentists wore a mask during dental treatment.

## 5. Conclusion

Dental anxiety likely predicts dental caries and may be a risk factor thereof. This study identified a significant difference between dmft/DMFT values and dental fear. In addition, children have strong preferences regarding the appearance of their dentist and dental clinics. To promote dental health, all members of the dental profession must be aware of patient perceptions, preferences, and fears in order to meet patient needs and provide quality care in a manner that is comforting and reduces anxiety.

## Figures and Tables

**Table 1:** Summary of the responses of the children to their previous dental experience.

Questions	Response	Number (%)
Have you been to a dentist before?	Yes	185 (92.5)
	No	15 (7.5)

How did you feel during dental treatment?	Liked it	143 (71.5)
	Do not like it	42 (28.5)

Is there a physician in your family?	Yes	13 (6.5)
	No	187 (93.5)

How did your sibling feel when he/she visited a dentist	Liked it	52 (26)
	Did not like it	148 (74)

**Table 2:** Summary of the perceptions of children of their dentists.

Questions	Response	Number (%)
Do you prefer to be treated by a male or female dentist?	Male	16%
	Female	84%

Which outfit do you prefer?	Colored coat	76.5%
	White coat	23.5%

Which dentist do you prefer?	No dental protection	20%
	Protective glasses	1%
	Mask	70.5%
	Mask and protective glasses	8.5%

**Table 3:** Distributions of dental fear and perception of the dental clinic according to age.

	Age
	Mean ± SD
Dental fear	
Yes	8.64 ± 1.54
No	9.69 ± 1.36
* P*	0.001**
Perception	
Undecorated clinic	9.74 ± 1.58
Decorated clinic	9.31 ± 1.44
* P*	**0.082**

Student's *t*-test **P < 0.01.

**Table 4:** Distribution of dmft and DMFT scores according to gender and dental fear.

	dmft	DMFT
	Mean ± SD (median)	Mean ± SD (median)
Gender		
Female	3.45 ± 2.41 (4)	1.36 ± 1.34 (1)
Male	3.59 ± 2.48 (4)	1.34 ± 1.25 (1)
* P*	**0.826**	**0.997**
Dental fear		
Yes	4.87 ± 2.37 (5)	1.11 ± 1.43 (0)
No	3.03 ± 2.29 (3)	1.44 ± 1.23 (1)
* P*	0.001**	0.044*

Mann-Whitney *U* test *P < 0.05, **P < 0.01.

**Table 5:** Perception of dentist gender according to the gender of the children.

	Girls	Boys	*P*
	*n* (%)	*n* (%)	
Preferred dentist			
Female	87 (88.8%)	81 (79.4%)	**0.071**
Male	11 (11.2%)	21 (20.6%)	

Chi-square test.
